# 106 What health resources do older adults find meaningful for participation in organized sport?

**DOI:** 10.1093/eurpub/ckae114.193

**Published:** 2024-09-26

**Authors:** Helena Ericson, Susanna Geidne, Mikael Quennerstedt

**Affiliations:** Örebro University, Örebro, Sweden; Örebro University, Örebro, Sweden; GIH Stockholm, Stockholm, Sweden

## Abstract

Physical activity represents one of the most beneficial strategies for people of all ages to retain an overall health. Regardless of the numerous benefits of regular physical activity older adults represent one of the least active groups in society. Although there are groups of older adults that meet the PA recommendations, still there remains a great deal of room for improvement not the least within organised sports.

Current knowledge on sports for older adults often focus on matters of how to avoid physical inactivity and overcome barriers to participation and thus understanding what causes illness rather than what promotes health. Literature also exists on the reasons why older adults drop out of sports and organized physical activities, even though it is sparse. Hence, even if this research is important in terms of understanding inactivity and non-participation, less is known about why old people continue to be physically active.

Against this backdrop, it is essential to investigate older adults who are physically active on a regular basis and what we can learn from them. A reason for targeting already physically active persons is that there is a lot to be learned from people who have the routines and habits needed to be active as an older adult.

The purpose of this project is to explore older adults’ experiences of participation in organized sports. In the project we use a health-promoting perspective focusing on sports clubs as a setting. The research questions that this project address are: What is experienced as meaningful in older adults’ sport participation? and What characterizes older adults’ participation in organized sports?

In the study we will investigate sports for older adults from a health-promoting perspective using an extensive data material of 4 837 older adults over 60 years participating in Swedish sports clubs. The sample included more than 1000 sports clubs with a large variation.

Previous results where the same questionnaires were used concluded that there were differences between what men and women as well as different ages experienced as meaningful in relation to the physical initiatives.

